# Real Talk: Conversations on HIV with Black Heterosexual Men in Healthcare Settings

**DOI:** 10.1007/s10900-024-01388-9

**Published:** 2024-08-10

**Authors:** Rakira Urquhart, Mackenzie Adams, Shawtaabdee Chakraborty, Jade C. Burns

**Affiliations:** 1 School of Health and Human Sciences, University of North Carolina at Greensboro, 1408 Walker Ave 437 Coleman Bldg, Greensboro, NC 27412 USA; 2 School of Public Health, University of Michigan, 1415 Washington Heights, Ann Arbor, MI 48109 USA; 3 College of Human Medicine, Michigan State University, 15 Michigan St, NE Grand Rapids, MI 49503 USA; 4 School of Nursing, University of Michigan, 400 North Ingalls Street Room 3175, 400 NIB, Ann Arbor, MI 48109 USA

**Keywords:** Black heterosexual men, HIV, AIDS, Men who have sex with Women, Reproductive health

## Abstract

Nearly half of heterosexually transmitted human immunodeficiency virus (HIV) infections occur among Black men in the United States. Yet Black heterosexual men (BHM) are largely ignored in HIV programming, policy implementation, and research. This study explores how masculinity, mental health, and socioeconomic factors such as income, education, and insurance (e.g., enrollment and coverage) correlate with the likelihood of BHM having important conversations surrounding HIV with their healthcare providers and family members. Conversations among social networks (e.g., peers, family, and neighbors) create an opportunity to increase comfortability while discussing HIV-related topics around condom use and testing. This study used a cross-sectional survey design and convenience sampling (*N* = 279) to recruit participants from a community-academic partnership involving a Federally Qualified Health Center (FQHC) in Detroit between June 1, 2020, and July 31, 2020. Descriptive statistics were used to report demographics, socioeconomic information, and sexual health-related behavior variables. Spearman’s correlation test was used to report bivariate correlations between predictor and outcome variables. 49.3% of the study participants disclosed having ever talked to a healthcare provider about HIV/acquired immune deficiency syndrome (AIDS), and 40.9% disclosed having ever talked to a family member about HIV/AIDS and sexually transmitted infections (STIs). The results from this article highlight potential barriers that may inhibit BHM from engaging in conversations about HIV with their healthcare providers and family members. It is important to include BHM in future research that focuses on HIV prevention and education to support community leaders and clinicians who work to address these disparities.

## Introduction

Over the past 40 years, the HIV/AIDS epidemic in the United States (US) has progressed in prevention, care, treatment, and evidence-based interventions [5, 19, 25]. However, it continues to be a concern in socially marginalized and disenfranchised populations such as communities of color [5, 25]. More specifically, HIV continues to disproportionately affect Black Americans, with 42% of all new HIV cases occurring among Black adults [6, 11, 17]. Black Americans, making up 12% of the US population, are a racially marginalized group that faces health disparities, often amplified by stigma and social determinants of health like economic status or attained education [9, 18, 28]. Reducing HIV- related stigma and discrimination can be achieved by supporting community efforts to address misconceptions that negatively impact HIV outcomes [28]. Also, decreasing misconceptions about HIV can be achieved by securing resources that are focused on populations who need it the most, especially Black gay, bisexual men, transgender people, people who use substances, sex workers, and immigrants in addition to Black heterosexual men [28]. Furthermore, it is estimated that 48% of new heterosexually transmitted HIV infections occur among Black men and this population has seen a 76% increase in transmission rates from 2005 to 2016 [11, 22]. Despite this disparity, BHM remains out of the narrative for HIV programming, policy implementation, and research within the United States [6, 11].

Several factors contribute to the continued prevalence of HIV among this population, including masculinity, lack of testing and screening, lack of awareness of HIV status, and social determinants of health such as transportation to a healthcare center, stigma, and health insurance [2, 7, 11]. A set of key attributes and principles offered by manhood play a significant role in showing distinct gendered, racialized, and class-based principles that are prioritized by men [26]. Sense of masculinity varies based on the individual and can affect interactions related to sexual health discussed in a social setting [14]. Challenges associated with accessing healthcare are related to help-seeking practices (e.g., stigma within the community, speaking with peers, family, or providers about health concerns) therefore, it is important to understand the role of masculinity and manhood in contributing to health disparities faced by Black men [4, 26].

Education, economic status, and environment can impact the desire to engage in precautionary measures like seeking health information or getting screened [8, 14]. Research has also shown that structural factors and poor patient-provider interactions may shape the quality of and access to health care [1]. For example, factors such as a lack of trust in the healthcare system may explain racial disparities regarding the HIV epidemic among Black Americans [3]. Interventions that direct efforts at clinicians and include training about taking sexual histories and the necessity of HIV testing are needed to effectively increase access to HIV testing and other relevant services (e.g., PrEP and Human Papilloma Virus vaccination) for BHM [16]. Taken together, these factors are essential because the lack of accessibility and affordability to health care may deter young Black men (YBM) from receiving adequate care regarding HIV prevention and treatment [2, 8]. Furthermore, data show that men who experience symptoms of mental illness are less likely to engage in safe sex behaviors [23]. However, comprehensive treatment plans that focus on mental health and HIV and sexually transmitted infection (STI) interventions have been found to be beneficial for this population, as evidence by the association between not being tested for HIV and depression, poverty, and lack of health care access [23, 29].

HIV risk reduction and prevention research among heterosexual men has been largely understudied, as there has been an emphasis within research and the literature on men who have sex with men, people who inject drugs, and heterosexual women [8, 32]. To promote inclusivity within HIV healthcare, there is a need to examine external factors in HIV transmission among BHM. Therefore, this study aims to explore how masculinity, mental health, and socioeconomic factors such as income, education, and insurance (i.e., enrollment and coverage) correlate with the likelihood of BHM having important conversations surrounding HIV with their healthcare providers and family members.

## Methods

The data were collected from a community-academic partnership involving a Federally Qualified Health Center (FQHC) that provides primary health care to about 15,000 patients yearly in Detroit. Most of the patients served (82%) are Black or African American. A cross-sectional survey design was used in this study, and convenience sampling was used to enroll participants between June 1, 2020 - July 31, 2020 [7]. Of the 485 survey responses, 279 met the inclusion criteria. This study was reviewed and determined to be exempt from requiring ethical approval by an Institutional Review Board.

Participants were recruited from health centers, community organizations, in-person contact, and social media. The survey was emailed to enrollees via an Eventbrite follow-up email after attending a virtual health event in June 2020. A second round of recruitment was conducted to increase survey responses. The survey was made available to the public on Instagram, Facebook, Twitter, and UMHealthResearch.org (resources used to connect researchers to volunteers). The survey was marketed to Black or African American men all over the United States. Information about the purpose and eligibility criteria of the study was included in the advertisement to attract more respondents.

Participants were eligible for the study if they documented themselves as (1) 18 years or older, (2) Black or African American, (3) male, and (4) heterosexual. Individuals were excluded if they reported that they did not reside in the United States. The survey’s primary goal was to evaluate and collect data on Black men’s attitudes and experiences during the early COVID-19 pandemic and generate measurable data on the subject. This dataset was selected because it focuses on Black, heterosexual men and includes measures on sexual health behaviors, including conversations about HIV with healthcare providers and family members. The data was collected during a critical time for the Black community and adds to existing literature that addresses health access for Black heterosexual men. Further, the aim of this paper aligns with the National HIV/AIDS 2022–2025 Strategy to decrease the incidence of HIV cases among all communities, especially priority populations, including Black men. This will help providers who care for patients at risk or living with HIV.

### Measures

Basic demographic (e.g., age, race, gender, country of birth) information was collected. Participants were asked about various socioeconomic factors using the following questions: 1) “Annual income before taxes?”; 2) “Marital or Partnership status?”; 3) Do you have health insurance?” *yes or no*. The following measures within the survey were used to describe sexual risk behaviors and testing (e.g., HIV/STIs) and depression.

### Sexual Health-Related Behaviors

Participants were asked to say “yes or no” to the following questions: 1) “Have you ever had any type of sex?”; 2) Have you ever talked to a healthcare provider about HIV/AIDS?”; 3) “Have you ever talked to a healthcare provider about condoms?”; 4) Have you ever talked about HIV/AIDS or STIs with a family member?”; Participants who answered yes to the previous item were asked to select who in their family: *parent*,* sibling cousin*,* aunt or uncle*,* or other*.

### Substance Use and Mental Health

Participants were asked about substance use and frequency of problems with mental health via the following two items: (1) “Have you smoked or had an alcoholic beverage in the last 30 days?” *yes* or *no*; (2) “Have you ever used marijuana or hashish?” *yes* or *no*. The survey asked, “How often do you feel hopeless?” rated on a 6-point scale from never to very frequently, which was adapted from the Patient Health Questionnaire 9 (PHQ-9) (Kroenke, Spitzer & Williams, 2001).

### Statistical Analysis

Descriptive statistics (frequencies, medians, and quartiles) were used for reporting demographics, socioeconomic information, and sexual health-related behavior variables. Spearman’s correlation test was used to report bivariate correlations between predictor (i.e., SES, health insurance, mental health, substance use) and outcome variables (i.e., having ever talked to a health care provider or family member about HIV/AIDs or STIs). This was based on non-normative data distribution and a two-tailed significance test (α = 0.05). Analysis was performed using SPSS v28.

## Results

Table 1 presents the sample characteristics (*N* = 279). The median age was 30 years old, most (55.6%) reported having an annual income of $30,000 or less, most were married or partnered (66.4%), and most (74.5%) had some form of health insurance coverage (including public and/or private insurance). Table 2 displays the descriptive statistics for the sexual health variables from the survey. Approximately half (49.3%) of participants reported having ever talked to a healthcare provider about HIV/AIDS, and less than half (40.9%) reported having ever talked to a family member about HIV/AIDS and STIs.

**Table 1:** Sample characteristics (*N* = 279)

Characteristic	*n*	% or median (quartile 1, quartile 3)
Age (in years) (*n* = 279)	279	30 (27, 37)
Annual income (before taxes) (*n* = 278)		
None	22	7.9
$1–10,000	22	7.9
$10,001–20,000	42	15.1
$20,001–30,000	68	24.4
$30,001–40,000	52	18.6
Over $40,000	72	25.8
Education level (highest completed)		
Less than 8th grade	10	3.6
9th -11th grade	30	10.8
High school graduate	50	17.9
Associate degree	78	28.0
Bachelor’s degree	63	22.6
Some graduate school	14	5.0
Graduate school or terminal degree	34	12.2
Married (or partnered) (*n* = 277)	184	66.4
Health insurance coverage (*n* = 278)		
Any (public and/or private)	207	74.5
None	71	25.5

**Table 2:** Sexual health-related behaviors among a sample of US black men (*N* = 279)

Variable	*n*	%
Ever had any type of sex (*n* = 278)	252	90.6
Had vaginal or anal sex without using condom in past 12 months (*n* = 276)		
Very frequently	29	10.5
Frequently	55	19.9
Occasionally	67	24.3
Rarely	39	14.1
Very rarely	48	17.4
Never	38	13.8
Ever been told by a doctor or nurse that you had a sexually transmitted disease	56	20.1
Ever talked to a healthcare provider about HIV/AIDS (*n* = 278)	137	49.3
Ever talked to a healthcare provider about STIs (*n* = 278)	157	56.5
Ever talked to a healthcare provider about condoms (*n* = 278)	142	51.1
Ever talked about HIV/AIDS or STIs with a family member	114	40.9
If yes – who (participant could select more than one)		
Parent	91	79.8
Sibling	65	57.0
Cousin	28	21.9
Aunt or Uncle	37	32.5
Other	82	71.9

As represented in Table 3, age, annual income, education level, substance use (smoking, alcohol, marijuana, or hashish), and more frequent feelings of hopelessness were not correlated with having ever talked to a healthcare provider about HIV/AIDS. Being married or partnered (r_s_: 0.17, *p* = 0.004) and having health insurance (r_s_: 0.20, *p* = 0.001) were positively correlated with having ever talked to a healthcare provider about HIV/AIDS.

**Table 3:** Factors correlated with ever having talked to a healthcare provider about HIV/AIDS in a sample of US black men (*N* = 279)

	Ever talked to a healthcare provider about HIV/AIDS		
	*n*	*r*_s_	*p*-value
Age (years)	278	0.01	0.87
Annual income (before taxes)	277	0.01	0.93
Education level (highest completed)	278	0.13	0.93
Married (or partnered)	276	0.17	0.004
Covered by health insurance	277	0.20	0.001
Smoked or had an alcoholic beverage in the last 30 days	278	-0.02	0.79
Ever used marijuana or hashish	278	-0.003	0.96
Feelings of hopelessness (never to very frequently)	278	-0.07	0.22

Spearman bivariate correlation, nonparametric, two-tailed test of significance

Age and substance use (smoking, alcohol, marijuana, or hashish) were not correlated with having ever talked about HIV/AIDS or STIs with a family member (see Table 4). Factors that were positively correlated with having ever talked about HIV/AIDS or STIs with a family member were having a relatively higher income (r_s_: 0.22, *p* < 0.001) a higher level of education completed (r_s_: 0.26, *p* < 0.001), being married or partnered (r_s_: 0.19, *p* = 0.001), and having health insurance (r_s_: 0.19, *p* = 0.002). More frequent feelings of hopelessness (r_s_: -0.19, *p* = 0.001) were negatively correlated with having ever talked about HIV/AIDS or STIs with a family member.

**Table 4:** Factors correlated with ever having talked about HIV/AIDS or STIs with a family member in a sample of US black men (*N* = 279)

	Ever talked to a healthcare provider about HIV/AIDS		
	*n*	*r*_s_	*p*-value
Age (years)	278	0.01	0.87
Annual income (before taxes)	277	0.01	0.93
Education level (highest completed)	278	0.13	0.93
Married (or partnered)	276	0.17	0.004
Covered by health insurance	277	0.20	0.001
Smoked or had an alcoholic beverage in the last 30 days	278	-0.02	0.79
Ever used marijuana or hashish	278	-0.003	0.96
Feelings of hopelessness (never to very frequently)	278	-0.07	0.22

Spearman bivariate correlation, nonparametric, two-tailed test of significance

## Discussion

This study provides insight into Black heterosexual men having conversations about HIV/AIDS and STIs with their healthcare providers and family members, and how socioeconomic factors (e.g., income, education, health insurance) and poor mental health may limit these conversations’ ability to occur.

Annual income, education level, and health insurance are factors that positively correlate with ever having talked to a family member about HIV/AIDS or STIs. At many healthcare facilities in the U.S., health insurance is necessary to receive access to quality care without patients being at risk of having unmet healthcare needs, including recommended screening tests [15]. Offering testing upfront and understanding patients’ needs, such as health insurance, may happen early on if these conversations occur among family members and trusted clinicians [27]. Documenting consistently in the electronic medical record about sexual history and taking thorough sexual records may inform clinicians on how to provide information about transmission, prevention methods, and overall sexual health [24].

It is also important to consider popular community gathering locations to reach as many people as possible with the intervention. Community organizations have the potential not only to help educate Black heterosexual men about the importance of HIV testing but also to address known factors that correlate with not talking about HIV at all [31]. If trust exists between healthcare providers, community leaders, and Black men individually, the conversation may help to alleviate stress from stigma and emphasize the importance of knowing HIV status [12].

It is necessary for clients to show up for appointments, but that is just the beginning. Presence alone does not guarantee that conversations about HIV will take place. Providers should address mental health challenges to ensure patients are in a space to prioritize their sexual health. Conversations about HIV can be difficult if stress, depression, or anxiety are overwhelming [21]. The quality of conversation may be improved by clinicians using a standard health screening tools to address concerns that may create barriers to care, such as health insurance and mental health care [13].

Additionally, our findings identify feelings of hopelessness negatively correlated with having a conversation about HIV with a family member. Training and leaning on community leaders to facilitate discussions as the first point of contact may address stigma and other barriers in discussing testing, screening, and engaging in risky behaviors [31]. That way, when men reach a clinical setting, HIV and sexual health behaviors are not new, and they feel more confident discussing with people outside of their intimate circles. Implementing a social support group and training members of community-based organizations as mental health professionals could positively impact stress and anxiety among BHM [10].

### Limitations

The study utilized a cross-sectional survey design, which limits the ability to speak to direct cause-and-effect relationships of the results. Limited questions were asked about sexual health behaviors; future surveys should include items from the Sexual Health Awareness Scale to assess attitudes, awareness, and practices regarding sexual health [20]. Moreover, adding questions about mental health from the Mental Health Quality of Life Questionnaire (MHQoL) may provide a clearer assessment of participants’ mental health status [30]. Data were primarily collected in Detroit during the peak of the COVID-19 pandemic using convenience sampling. Therefore, it may not be representative of the population. Future surveys should seek a larger nonrandom sample. A beta test to evaluate survey length, readability, and item order may be beneficial before disseminating to the public.

### Implications

Future clinical efforts should prioritize building rapport with potential patients before meeting them in a health clinic. This can be achieved through collaboration with grassroots organizations and participation in established community events, such as church gatherings and health fairs. The key to success is to make the information available to clinicians in these settings, which may foster trust and familiarity, leading to patients being more receptive to having sensitive conversations about sexual health beyond their immediate peer group. Additionally, patients receiving extra support before and after clinic visits may have greater confidence in making informed sexual health decisions.

Including questions about mental health and other life experiences, in addition to sexual histories, gives a more complete picture of how to help the patients best. By understanding patients’ lived experiences, healthcare providers can address fears and misconceptions about HIV in a more tailored manner, leading to improved patient outcomes. Furthermore, continuous education and anticipatory guidance need to be offered during healthcare visits. Practices that fail to provide such support contribute to gaps in care, reinforcing the importance of incorporating ongoing education, and guidance into healthcare services [24]. Future research should prioritize including high-quality data on provider-patient interactions among heterosexual Black men. Such research will offer valuable insights into the unique needs of this population and help distinguish how gender and sexual orientation must be considered separately in healthcare interactions. These factors can change the conversation based on lived experiences and dominating perceptions about HIV in society.

## Conclusion

This study identifies potential barriers to Black heterosexual men having conversations regarding HIV/AIDs and STIs with their healthcare providers and family members. The findings of this study support the need for tailored community interventions that aim to increase BHM participation in essential conversations about HIV. When developing solutions, healthcare providers, researchers, and community leaders should consider socioeconomic and mental health barriers to address HIV and STI concerns. To improve BHM engagement in HIV and STI prevention and education, it is essential to address barriers such as low socioeconomic status and a lack of health insurance because they can impact accessing healthcare and health information. As Black heterosexual men continue to remain impacted by new incidences of HIV diagnoses, it is important to prioritize this population in future research when focusing on HIV education and prevention.
